# Asylum-seeking children with resignation syndrome: catatonia or traumatic withdrawal syndrome?

**DOI:** 10.1007/s00787-019-01427-0

**Published:** 2019-11-01

**Authors:** Anne-Liis von Knorring, Elisabeth Hultcrantz

**Affiliations:** 1 Department of Neuroscience, Child and Adolescent Psychiatry, Uppsala University, 75185 Uppsala, Sweden; 2 Division of ORL, Department of Clinical and Experimental Medicine, Linköping University, Linköping, Sweden

**Keywords:** Resignation syndrome, Traumatic withdrawal syndrome, Catatonia, Asylum seeking, Children, Hopelessness, Fear

## Abstract

In the beginning of the 2000s, an increasing number of asylum-seeking children in Sweden fell into a stuporous condition. In the present study, we report 46 consecutive children with the most severe form of this illness where the children were unable to give any response at all, did not react to pain, cold or touching, could not be supported to sit or stand on their feet, could not do anything when requested, and in most cases had enuresis/encopresis. A minority of the children came from war zones (*n* = 8, 17.4%). A majority belonged to an ethnic or religious minority (*n* = 32, 69.6%) in their homeland and almost all were persecuted (*n* = 43, 93.5%). All had either experienced violence themselves or had witnessed or heard about violence against close family members. The age of onset of the first symptom of illness for boys was 11.2 years [CI 9.6–12.8], for girls 11.8 yrs.[CI 10.4–13.2], and the age for falling into stupor for boys was 12.9 years [CI 11.6–14.1] years and was the same for girls, 12.9 years [CI 11.6–14.2] years. Girls tended to have depression before entering the stuporous condition, while the boys tended to have PTSD first (Chi-square = 3.73, *p* = 0.054). A majority of the children had one (*n* = 13, 28.3%) or both parents (*n* = 14, 30.4%) suffering from mental or severe physical disorder. It is discussed whether the presented condition is a separate entity or if the syndrome should be regarded as a variant of catatonia, and whether benzodiazepines should be tried.

## Introduction

In the beginning of this century, an increasing number of asylum-seeking children in Sweden went into a so far unknown stuporous condition. This condition was not recognized by pediatricians and child psychiatrists, although it had already been described in 1958 by the Swedish child and adolescent psychiatrist Anna-Lisa Annell as a very rare disorder occurring mostly after severe psychological trauma [1]. The Swedish Association of Child and Adolescent Psychiatry screened all child and adolescent psychiatry clinics in Sweden in 2004 and found that 424 refugee children and adolescents 0–20 years had been treated from 2003 to June 2005 because of reduced communication, motor skills and ability to carry out daily routines. Approximately, 1 out of 3 of the 425 were fed by means of a nasogastric tube as they were unable to eat or drink. Those children who needed a nasogastric tube were also mute and were laying down. They were not moving at all, were hypotonic, and totally blocked from the environment without any formal or emotional contact with people in their environment. Their eyes were closed all the time. The children did not react on touch, sound, pain or cold. Most had enuresis and encopresis. Some parents managed to take them on a wheelchair regularly to the toilet. Those symptoms were later classified as symptoms “grade 2” of this unknown disease, which in the beginning was called “apathy”, and later on when more knowledge and experience had been gathered: “resignation syndrome”. If the children could show some response when spoken to, walk with support, do things when requested and could be spoon fed, the condition was classified as “grade 1” [2]. On July 1, 2014, the diagnosis of *resignation syndrome* was included in the Swedish version of ICD-10 with classification, ICD-10-SE; F 32.3A. Since then, this diagnosis has been reported in the register of the National Board of Health and Welfare, making studies in epidemiology possible.

Very few children with the same or similar symptoms have been reported from other European countries [3]. Recently, however, there have ben reports from Australia of a number of refugee and asylum-seeking children with a syndrome very similar to the resignation syndrome among those who had been on the Island Nauru for several years [4].

### Aims of the study

The aims of the present study were:

1. to describe the background of the children with a resignation syndrome with regard to traumatic experiences, parental mental health and family situation;
2. to describe the course of mental illness before the onset of the resignation syndrome;
3. to discuss the classification of the syndrome.

## Methods

46 consecutive children with RS grade 2 (*n* = 46, 22 boys, 24 girls) were included during the years 2010 to 2018. One child treated during 2004 at the Department of Child and Adolescent Psychiatry, Uppsala University Hospital, was also included. They all had been examined by one or both of the authors more than once. Three families had two children with resignation syndrome. Since both siblings with resignation syndrome were included in the series, in total six children had a sibling with the same symptoms. All families were in the asylum process. For details see Table 1.

**Table 1:** Demographic information from 46 children with resignation syndrome

	Boys *n* (%)	Girls *n* (%)	*p* value	Total *n* (%)
Sex	22	24	n.s	46
Oldest sibling or only child	16 (72.3)	9 (37.5)	0.02	25 (54.3)
Minority, ethnic, religious	14 (63.6)	18 (75.0)	n.s	32 (69. 6)
Denied residency	16 (72.7)	22 (91.7)	n.s	38 (82.6)
No final decision	6	2	n.s	8

### Examination

All children were examined at least two times. Information on all previous traumas in homeland, during the escape, and in Sweden was asked from the parents. As the children did not communicate, the parents also were systematically asked about earlier mental and somatic symptoms. Medical records from general practitioners, pediatric and child psychiatric departments were studied, as well as medical statements from physicians and social workers obtained. Diagnoses according to ICD-10 research criteria of mental disorders were used [5]. All diagnoses of mental disorders were made by the same trained, experienced child psychiatrist (ALvK).

The Swedish version of Children’s Global Assessment Scale (C-GAS) was used to retrospectively estimate the function of the children at arrival to Sweden and later on at all examinations [6].

The children’s current loss of functions was described in terms of capacity to communicate, move, eat and drink and carry out daily routines. The capacity to carry out any such tasks was rated on a modified scale ranging from normal (0) to total loss of function (− 4 to − 6), meaning that a lower number indicates a more severe condition. In this scale, the lowest possible score was − 39 [7]

### Statistics

In the presentation of the material, descriptive statistical methods have been used, i.e., mean, range and [CI].

When distributions are compared, the Chi-square test with one degree of freedom was used. Differences between means were tested by means of the Mann–Whitney *U* test due to non-normal distributions. All analyses were done using SPSS 24.

## Results

### Subjects

Table 1 demonstrates the demography of the 46 included children. Eight of them came from ex-Yugoslavia (3 boys, 5 girls), 5 from Iraq/Syria (1 boy, 4 girls), and 33 from ex-Soviet Union/Russia (18 boys, 15 girls). All children except one (*n* = 45) were taken care of by their parent(s) at home with support from health and social services, after an initial hospitalization of 3–10 days. One child had only been treated in hospital. They all had the most severe form of the resignation syndrome (grade 2) and all except two were tube fed, when examined for the first time. One had been tube fed prior to our first examination, and the other after. A minority of the children studied came from war zones. A majority belonged to an ethnic or religious minority (*n* = 32, 69.6%) in their homeland, Uighurs (*n* = 8, 17.4%), Romani (*n* = 7, 15.2%), Yezidis (*n* = 6, 13.0%), Armenians in Russia/Ukraine (*n* = 6, 13.0%), and others (*n* = 5, 10.9%). Almost all had been persecuted in the homeland. Fourteen (30.4%) had parents, who fled for political reasons. Only one boy was unaccompanied by his parents, who probably were dead. Six boys came together with only one parent, two fathers and four mothers. Three of these boys were the only children.

### Trauma

Most of the children had in their homeland been forced to witness violence, rape or killing, and/or threats against a close family member or had been victims themselves. Thirty-seven children (80%) had been exposed to such experiences repeatedly. There was no statistically significant difference between boys and girls with respect to the type of traumatic event(s). For details see Table 2.

**Table 2:** Background of trauma for the 46 asylum-seeking children with resignation syndrome

Type of trauma	Boys *n* = 22 (%)	Girls *n* = 24 (%)	*p* value	Total *n* = 46 (%)
War zone	5 (22.7)	3 (12.5)	n.s	8 (17.4)
Persecuted in homeland	20 (90.9)	23 (95.8)	n.s	43 (93.5)
Victim of violence	11 (50.0)	14 (58.3)	n.s	25 (54.3)
Witnessed violence inflicted on family member	17 (77.3)	20 (83.3)	n.s	37 (80.4)
Victim of rape	0	1	n.s	1
Forced to witness mother being raped	5 (22.7)	6 (25.0)	n.s	11 (23.9)

### Pathogenesis

44 (95.6%) children suffered from posttraumatic stress syndrome (PTSD) and/or a depressive episode, which developed to resignation syndrome. There was a tendency for girls to have depression before resignation syndrome, while the boys first had PTSD (chi^2^ = 3.73, *p* = 0.054). Three children had made suicide attempts, and another three had communicated suicidal ideation to their parents. Two children reacted immediately with severe anxiety after being informed about deportation by a clerk at the Migration Board and within a few days both were not eating, moving, hypotonic, and totally blocked from the environment without any formal or emotional contact (Table 3).

**Table 3:** Diagnoses of mental disorders according to ICD-10 prior to the onset of resignation syndrome (retrospectively evaluated)

	Boys *n* = 22 (%)	Girls *n* = 24 (%)	Total *n* = 46 (%)
Depression prior to resignation syndrome	5 (22.7)	12 (50.0)	17 (37.0)
Posttraumatic stress disorder prior to Resignation syndrome	16 (72.7)	11 (45.8)	27 (58.7)
Secondary depression	8 (36.4)	4 (16.7)	12 (26.1)
Secondary posttraumatic stress disorder	0	4 (16.7)	4
Suicide attempt/communicated suicidal ideation	1	5 (20.8)	6 (13.0)
Severe acute stress reaction prior to resignation syndrome	1	1	2

### Onset

The two most common first symptoms of resignation syndrome the parents reported was fear in 11 cases (8 boys, 3 girls, chi^2^ = 3.59, *p* = 0.06) and reduced speech in 11 cases (6 boys, 5 girls, n.s). Sleeping problems were reported in nine cases (3 boys, 6 girls, n.s), depressive mood in five cases (2 boys, 3 girls, n.s), and withdrawal in five cases (3 boys, 2 girls, n.s). Loss of appetite was reported as the first symptom in two girls, weakness of the legs in two other girls, and irritability in one girl.

The age of onset of the first symptom was 11.5 years [CI 10.5–12.6], with no significant differences between boys and girls.

The time from first sign of mental illness to the development of the full resignation syndrome varied widely. The mean time for boys was 682 days [CI 230–1128], and 382 days [CI 232–531] for girls (n.s). A majority of the children went from grade 1 to grade 2 of the resignation syndrome in just a few days.

**Fig. 1:**
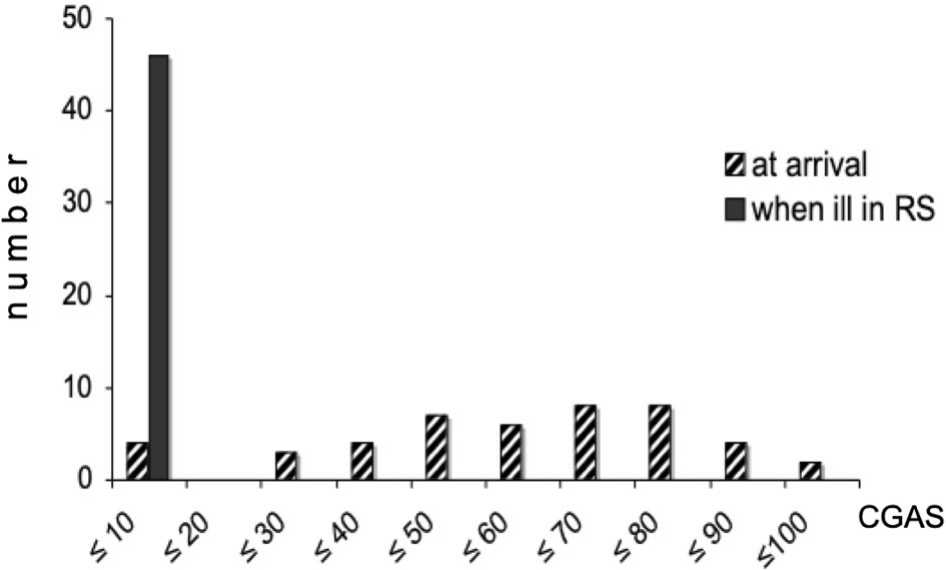
Children’s Global Assessment Scale of 46 asylum-seeking children with Resignation Syndrome (RS), evaluated retrospectively at arrival to Sweden (striped staples) and when ill, at first examination (filled staples)

Four children (3 boys, 1 girl) already suffered from resignation syndrome grade 2, and two children suffered from RS grade 1 (1 boy, 1 girl) when they arrived at Sweden. The children with grade 2 had the lowest level of functioning on the C-GAS at arrival and those with grade 1 were assessed with a C-GAS of 25. The C-GAS for all 46 children at arrival to Sweden (retrospectively assessed) and at our first examination is shown in Fig. 1.

### Family situation

A majority of the children had one (*n* = 13, 28.3%) or both parents (*n* = 14, 30.4%) suffering from mental or severe physical disorder. For details see Table 4.

**Table 4:** Health problems of the parents to the children with resignation syndrome

	Mother *n* = 46 (%)	Father *n* = 46 (%)
Trauma-related mental disorder	7 (15.2)	6 (13.0)
Depression	10 (21.7)	4 (8.7)
Alcoholism/antisocial personality disorder	0	4 (8.7)
Other mental disorder	2	0
Severe physical illness	3	5 (10.9)
Dead	2	5 (10.9)
Total	24 (52.2)	24 (52.2)

The 46 children with resignation syndrome had 86 siblings of whom 4 were diseased. Ten (12.2%) of the siblings suffered from trauma-related mental disorders, 13 (15.9%) from depression, 6 from resignation syndrome, 4 had a language disorder, 1 suffered from separation anxiety disorder, and 1 from enuresis. In total, there were 35 (42.7%) siblings with a mental disorder. Three boys were the only child and came to Sweden with just one parent. One boy was an unaccompanied minor with no siblings.

### Trigger

There were different triggers for the children to go into resignation syndrome: Most common was to fall into coma after having been present at the meeting with the Migration Board when informed of the negative decision and the coming deportation (every other child) or they had themselves opened and read the letter (in Swedish) of rejection without a parent’s support. Nine children (19.6%) had developed severe mental symptoms immediately after being a victim of violence in the home country and three children saw their mother try committing suicide. These children all had a progression of other symptoms prior to resignation syndrome. Three girls (all Yezidis), already with negative decisions, had repeatedly watched YouTube during August 2014 and later about how the Islamic State treated people/women in Syria and Iraq with faiths other than Islam. Three other children had a sibling (two) or mother (one) with resignation syndrome, three were victims of violence or harassment in Sweden, and two had been exposed to a police raid at home in Sweden as a triggering factor.

### Deportation

Three families had earlier applied for asylum in Sweden and been deported before any child was ill. When these families returned to Sweden, two children already suffered from resignation syndrome after new traumas. In the third family, the child fell ill after rejection of their new asylum application from the Migration Board.

Four other cases were deported to another EU country according to the Dublin agreement. Two of them were treated as inpatients in hospital together with one parent, and both improved. One was sent back to Sweden directly in connection with the deportation without entering the country because of the child´s severe illness. The fourth child was lost to follow-up.

Only one case with resignation syndrome was deported back to the homeland, where there was no knowledge about the condition. The father had to feed his sick child through the tube with baby formula he bought himself both during hospital stay and later at home. The tube clogged up and this family returned after 4 months. The child was then severely dehydrated and in a worse condition than when he left. Permanent residency permit was obtained after another 7 months and the child started to recover.

## Discussion

This is the first prospective study of a large number of children with resignation syndrome. This paper deals with the phase of their disease, when they have been mostly taken care of by their parents at home, and the whole family had lived in insecurity, already with negative decisions or waiting for the last decision.

The most important result is the finding that all asylum-seeking children who have developed resignation syndrome in Sweden, or were affected when they arrived, have been exposed to life-threatening traumas in their homeland, persecution and violence. Most of them belong to ethnic-suppressed minorities in their homeland. Since only six children had a sibling also with resignation syndrome, an individual vulnerability seems to be present apart from difficult, traumatic living condition. Almost all had a history of mental illness, depressive disorder and/or PTSD, which most often had begun in connection with a specific trauma. There were no significant gender differences with respect to the vulnerability to develop resignation syndrome.

The acute onset of the resignation syndrome was most often triggered by a negative decision from the migration authorities, when either the child read a negative decision in a letter (written in Swedish) and had to translate the content to the parents, and/or the child was required to be present when the negative decision was orally given to the family. In both those cases, the child, who usually understood Swedish best, was the first one to understand the negative decision, before the translation was made to the parents. Some children reacted immediately at that point with vomiting or other physiological symptoms. As it also was common that both parents and siblings suffered from mental disorder/distress, the possibility for support within the family was limited. The child who fell ill was usually the one who had been responsible in the family, who often acted as a translator (the oldest or only son), and/or most often the one who had been witnessed the most traumatic event in the home country (rape of mother, torture or killing of father).

The children’s reactions at the triggering moment were very similar to the described concept of learned helplessness known in many mammals [8]; when all hope for safety seems to be lost, in an acute fear/stress situation the individual goes into a catatonic state which is irreversible without intensive care. Another well-known concept among both mammals and birds is the acute fear reaction “freezing” or “play dead reaction” where the old part of the vagus nerve seems to be involved [9, 10]. The neurophysiological mechanisms behind RS have to be further studied, which also is planned.

Only a few children in our series came from war zones. Refugee families from war zones have so far got asylum in Sweden without extensive delay, and their children can therefore earlier start recovering from the traumas they have experienced without any more traumatic triggers.

It has been discussed whether the resignation syndrome is a separate, new entity, or if the condition should be regarded as a variant of pervasive refusal syndrome, dissociative stupor, depressive stupor or catatonia [11, 12]. According to the definition of pervasive refusal syndrome by Jaspers: the patients refuse actively and angry to acts of help and encouragement, and no other psychiatric condition could better account for the symptoms [13]. Patients with resignation syndrome are hypotonic, and according to ICD-10, dissociative stupor includes normal muscle tone, and those with dissociative stupor also react normally to loud noise and touch. Children with resignation syndrome do not react to any sensory stimulation, not even pain, and only a few refused for a short time actively and angry to acts of help in the early stage of resignation syndrome.

In the new ICD-11, both dissociative stupor and depressive stupor do not remain as special diagnoses [14]. Instead, the diagnosis is named catatonia associated with another mental disorder, as in DSM 5 [15].

No doubt, the children with resignation syndrome fulfill the DSM 5 criteria for catatonia. They present with 3 of the 12 specified criteria, i.e., stupor (no psychomotor activity, no reactivity to the environment), mutism (no or minimal verbal response), and negativism (not responding to external stimuli or instructions). However, the other nine specified criteria, catalepsy, waxy flexibility, posturing, mannerism, stereotypy, agitation, grimacing, echolalia or echopraxia, were not found in any of the cases or had been preceding symptoms.

Catatonia as a concept was originally introduced by Kahlbaum and was at that time seen as a separate entity linked to manic, depressive and psychotic disorders [16]. Later, Kræpelin [17] linked catatonia to dementia praecox and Bleuler described catatonia as a subtype of schizophrenia [18]. In the earlier versions of the ICD and the DSM classification systems, catatonia was usually linked to schizophrenia. However, Leonhard clearly linked catatonia also to affective disorders and anxiety disorders [19]. In 1997, Peralta et al. presented data to raise the possibility that catatonia might be a variant of mood disorder or a distinct entity [20]. This view was later introduced in the DSM 5. The process has been described by Luchini et al. [21]. It has also been demonstrated that catatonia is rather common in children and adolescents [22].

However, even if the resignation syndrome is regarded as a special variant of catatonia, many questions remain unanswered. Even if catatonia is regarded as a separate entity, a number of medical or neurological disorders may appear with a similar picture or may have an overlap with catatonia [23]. All children with resignation syndrome in this study showed the almost identical clinical picture. The patients included in the present series were not diagnosed with any medical or neurological disorders and none of them had any symptoms or signs of schizophrenia.

It has been argued by Shorter and Fink that catatonia is associated with fear and alarm, triggered by trauma. It has been linked to the animal defense of tonic immobility in a predatory environment [24]. In the children described, affective, fear, and severe trauma-related disorders are common [25]. The histories of the children in the present study are in line with such a view. A similar view has also been taken in Australia, where a disorder with the same background has been described and labeled traumatic withdrawal syndrome [4].

So far, there are no controlled studies of treatment options of catatonia in children, although prospective case series have been published. Benzodiazepines relieve the symptoms of catatonia [26]. It would be of interest to try such a treatment regimen in the asylum-seeking children with resignation syndrome in a systematic study, especially in the initial phase when the child is anyhow being treated in hospital. The present care by the parents at home with support of the health-care system, but without drugs, was earlier sufficient as long as the time for decision about permanent safety in the country was some months away [27]. However, now when many children have been laying in this condition for 3 years and more, a more active medical treatment policy is suggested.

## Conclusion

Resignation syndrome is always related to earlier severe traumas leading to PTSD or/and depression and has several catatonic features [28]. If classified as the retarded type of catatonia, a treatment trial with benzodiazepine is suggested.
